# A Selection Fit Mechanism in BMP Receptor IA as a Possible Source for BMP Ligand-Receptor Promiscuity

**DOI:** 10.1371/journal.pone.0013049

**Published:** 2010-09-28

**Authors:** Stefan Harth, Alexander Kotzsch, Junli Hu, Walter Sebald, Thomas D. Mueller

**Affiliations:** 1 Lehrstuhl für Physiologische Chemie II, Theodor-Boveri-Institut für Biowissenschaften der Universität Würzburg, Würzburg, Germany; 2 Lehrstuhl für Molekulare Pflanzenphysiologie und Biophysik, Julius-von-Sachs Institut der Universität Würzburg, Würzburg, Germany; Griffith University, Australia

## Abstract

**Background:**

Members of the TGF-β superfamily are characterized by a highly promiscuous ligand-receptor interaction as is readily apparent from the numeral discrepancy of only seven type I and five type II receptors available for more than 40 ligands. Structural and functional studies have been used to address the question of how specific signals can be deduced from a limited number of receptor combinations and to unravel the molecular mechanisms underlying the protein-protein recognition that allow such limited specificity.

**Principal Findings:**

In this study we have investigated how an antigen binding antibody fragment (Fab) raised against the extracellular domain of the BMP receptor type IA (BMPR-IA) recognizes the receptor's BMP-2 binding epitope and thereby neutralizes BMP-2 receptor activation. The crystal structure of the complex of the BMPR-IA ectodomain bound to the Fab AbD1556 revealed that the contact surface of BMPR-IA overlaps extensively with the contact surface for BMP-2 interaction. Although the structural epitopes of BMPR-IA to both binding partners coincides, the structures of BMPR-IA in the two complexes differ significantly. In contrast to the structural differences, alanine-scanning mutagenesis of BMPR-IA showed that the functional determinants for binding to the antibody and BMP-2 are almost identical.

**Conclusions:**

Comparing the structures of BMPR-IA bound to BMP-2 or bound to the Fab AbD1556 with the structure of unbound BMPR-IA shows that binding of BMPR-IA to its interaction partners follows a selection fit mechanism, possibly indicating that the ligand promiscuity of BMPR-IA is inherently encoded by structural adaptability. The functional and structural analysis of the BMPR-IA binding antibody AbD1556 mimicking the BMP-2 binding epitope may thus pave the way for the design of low-molecular weight synthetic receptor binders/inhibitors.

## Introduction

Bone morphogenetic proteins (BMPs) are secreted multifunctional signaling proteins that belong to the TGF-β (Transforming Growth Factor β) superfamily [1]. Members of the BMP subfamily play important roles during development, maintenance and regeneration of tissues and organs in almost all vertebrates and non-vertebrate animals [2], [3]. Their malfunction during the signalling process can lead to multiple diseases including skeletal malformations, cardiovascular and metabolic diseases, muscular disorders and cancer [4].

Signal transduction of TGF-β proteins is initiated by binding to two types of receptors named type I and type II [1], [5]. Both receptor classes share a cysteine-rich extracellular domain, a single transmembrane segment and a cytoplasmic serine/threonine-kinase domain. Upon receptor oligomerization, the type I receptor is phosphorylated at its intracellular glycine-serine-rich domain (the so-called GS box) by the constitutively active type II receptor kinase thereby activating the SMAD signalling cascade affecting transcription of responsive genes in the nucleus [6].

One hallmark of the TGF-β ligand-receptor interaction is its high promiscuity [7], with only seven type I receptors and five type II receptors being known for more than 40 ligands [1], [8]. Thus one receptor subtype usually interacts with several different ligands. Furthermore, various ligands have been shown to interact with different receptor chains of both type I and type II. For instance, BMP-2 recruits both type I receptors, BMPR-IA and BMPR-IB, into a binary complex with high affinity. A low affinity type II receptor (either BMPR-II, ActR-II or ActR-IIB) is then bound by the binary complex, thereby forming a heterotetrameric receptor complex [7].

In recent year, several crystal structures of BMPs bound to the extracellular domains of type I and type II receptors have been determined in recent years to unravel the molecular mechanisms underlying promiscuity and specificity [9], [10], [11], [12], [13], [14]. Three crystal structures of the extracellular (EC) domain of BMPR-IA in complex with BMP-2 are described, showing that binding and structure of BMPR-IA are highly conserved, although crystallisation conditions varied in all three cases [9], [11], [14]. Recently, the NMR structure of unbound, free BMPR-IA_EC_ was determined, showing that its core structure is largely superimposable upon the structure of the receptor bound to BMP-2 [15]. However, the binding epitope of BMPR-IA to BMP-2 differs markedly due to the absence of a short α-helix in the β4β5-loop of the free receptor. Importantly, the α-helical segment of BMPR-IA is in the centre of the BMP-2 binding epitope and carries the hot spot of binding, Phe85 and Gln86, for binding to BMP-2. Upon complex formation with BMP-2, a disorder-to-order transition occurs in the receptor, indicating an inherent flexibility of a main binding element. Similarly, BMPR-IB also seems to exhibit inherent flexibility in the ligand binding epitope, which in the case of binding to GDF-5 is used to generate ligand binding specificity [13].

Here we report the crystal structure of BMPR-IA_EC_ bound to the BMP-2 activity-neutralizing Fab AbD1556. Structural analysis showed that the β4β5-loop of BMPR-IA does not adopt an α-helical conformation as seen in the complex BMP-2:BMPR-IA_EC_, but rather exhibits an extended conformation similar but not identical to the NMR structure of BMPR-IA. Therefore, the formation of the α-helix in BMPR-IA's β4β5-loop seems to depend on the nature of the binding partner and is thus not “imprinted” during complex formation with a protein binding partner. Surprisingly, despite the differences in the structural epitopes of BMPR-IA for Fab- and BMP-2 binding, the same residues in BMPR-IA seem to be involved in recognition and binding to BMP-2 and AbD1556. Taken together, these results provide new and interesting insights for our understanding of protein-protein interactions and protein recognition.

## Materials and Methods

### Preparation of BMPR-IA_EC_ and selection of Fab fragments

The extracellular domain of human BMPR-IA (BMPR-IA_EC_) (amino acids 1–129 of the mature part, SWISSPROT entry P36894) and its variants were expressed as thioredoxin-fusion proteins as described [16]. Mutations were introduced by two-step PCR-based targeted mutagenesis. Wildtype and variant proteins of BMPR-IA were purified using an identical protocol, thus differences in the purification could possibly indicate BMPR-IA variants with non-native fold. Protein homogeneity was analyzed by reversed-phase HPLC chromatography, SDS-PAGE and mass spectrometry.

Recombinant Fab proteins were obtained from AbD-Serotech (Martinsried). Initially, eight antibodies against the extracellular domain of BMPR-IA were selected from panning of a HuCal phage library with biotinylated BMPR-IA_EC_ immobilized on a resin [17], [18], [19]. Antibodies were characterized by western blot, cell assays and interaction analysis. Two of these Fabs, AbD1556 and AbD1564, showed high nanomolar affinities for BMPR-IA and could neutralize BMP-2 activity in cell-based assays. Both Fab proteins used in this study contained a non-cleavable Strep-Tag at the C-terminus of the heavy chain (peptide sequence SAWSHPQFEK), which was not removed for crystallization.

### Crystallization of Fab-receptor complexes

A binary complex of AbD1556 bound to BMPR-IA_EC_ was formed by mixing AbD1556 with a 1.1-fold molar excess of BMPR-IA_EC_. The protein complex was purified by gel filtration using a Superdex200 HR10–30 column (GE Healthcare) and 10 mM HEPES pH 7.4, 150 mM NaCl. Fractions containing the antibody-receptor complex in equimolar stoichiometry were combined, and the protein solution was concentrated up to 16.4 mg ml^−1^ in 10 mM HEPES pH 7.4, 150 mM NaCl using ultrafiltration. Initial screening for crystallization conditions was performed using a sparse matrix setup obtained from successful crystallization conditions reported for antibody-protein complexes in the RCSB databank and several commercial screens (Hampton, MDL) [20]. Single crystals of the AbD1556:BMPR-IA complex could be grown by mixing 2 µl protein solution and 1 µl reservoir solution in hanging drop setups at room temperature over a reservoir solution of 100 mM Tris-HCl pH 7.0, 20% (w/v) PEG 8000 and 10% (w/v) glucose as cryoprotectant. Protein crystals grew to a final size of approximately 150×150×40 µm within 7 days. Crystal suitable for data acquisition diffracted to a resolution limit of up to 2.7 Å and had the monoclinic space group P2_1_ with unit cell parameters of a = 89.32 Å, b = 129.25 Å, c = 100.24 Å and α = γ = 90° and β = 92.27°.

### X-ray data acquisition and structure analysis

**Figure 1:**
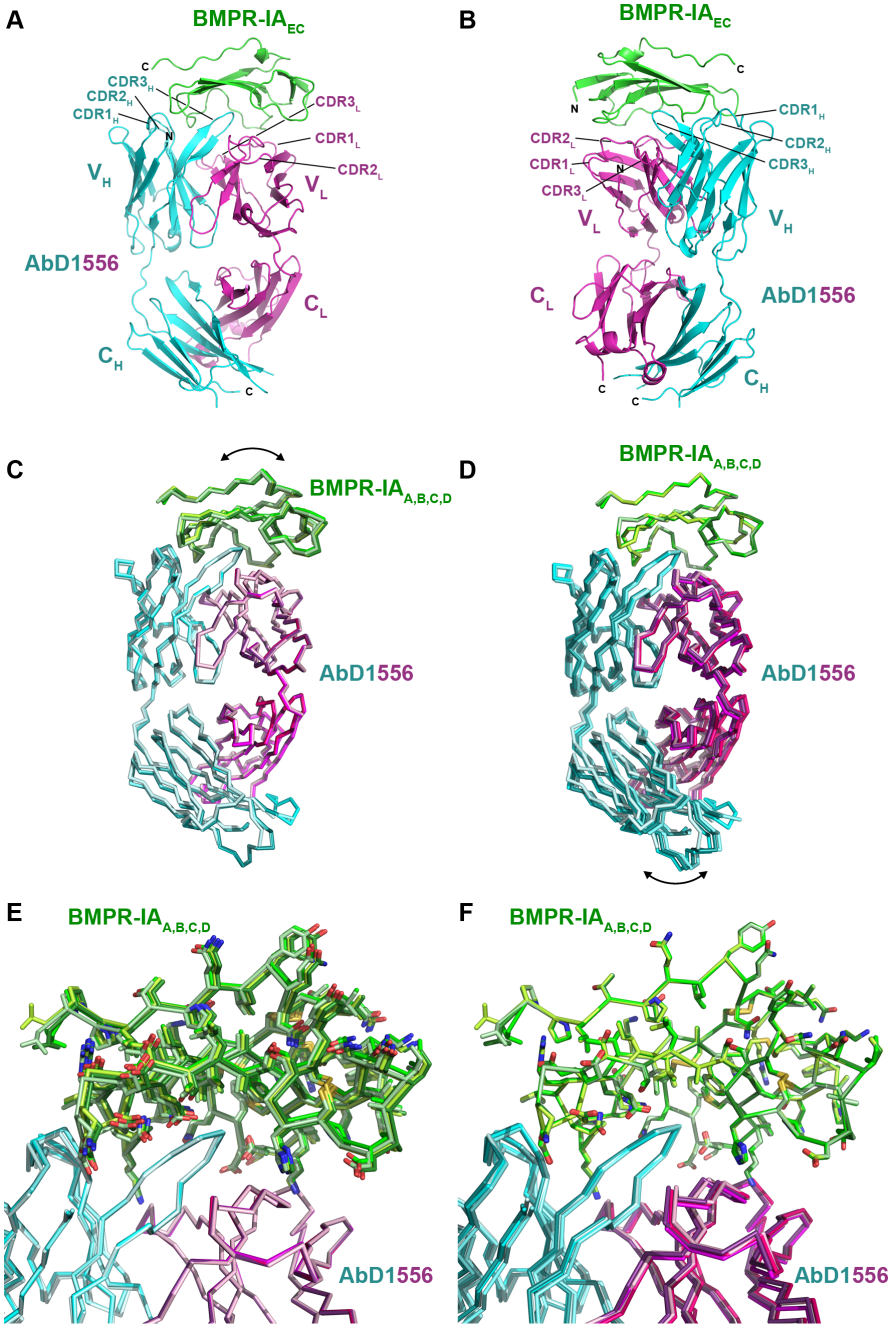
Architecture of the Fab:receptor complex of AbD1556:BMPR-IA_EC_. (**A**) Ribbon representation of the Fab:receptor complex of BMPR-IA bound to AbD1556. The receptor ectodomain (Pro34 - Val118) is shown in green, the heavy chain of AbD1556 is shown in cyan, the light chain of the Fab is marked in magenta. Complementary determining regions (CDRs), constant and variable regions are indicated. (**B**) As in (A) but rotated by 150° around the y-axis. (**C**) Structural alignment of the four Fab:BMPR-IA complexes of the asymmetric unit. The four structures of BMPR-IA_EC_ are shown in different shades of green, similarly the Fab heavy and variable regions of the four Fab molecules of the asymmetric unit are indicated in different shades of cyan and magenta. Only the Cα-trace is shown. Structural superposition was performed using all Cα-atoms of the four Fab molecules thereby showing slight, but significant differences in the location of the four BMPR-IA molecules (∼1 Å). (**D**) As in (C) but structural alignment was performed on the Cα-atoms of BMPR-IA. (**E**) As in (C) but side chains of the four BMPR-IA molecules of the asymmetric unit are shown, illustrating the positional shifts of the BMPR-IA molecules in the Fab binding site. (**F**) As in (E) but with structural alignment performed on the Cα-atoms of the four BMPR-IA molecules.

A native dataset of the Fab AbD1556-BMPR-IA_EC_ complex was acquired from a single crystal on a X-ray home source (X-ray generator Rigaku MicroMax007 equipped with Osmic VariMax HighRes optics and an image plate system RAXIS-IV++) at 100 K. Diffraction data were indexed and integrated using the software CrystalClear 1.3.6 (Rigaku). The dataset used has 95.3% completeness for the resolution range 30.4 to 2.7 Å and an R_sym_ of 0.08. Initial phasing was performed by molecular replacement using the structure of the human Fab PDB entry 1AQK as template and using the software Phaser [21]. Calculating the Matthew's coefficient indicated three or four Fab:receptor complexes per asymmetric unit (3 AbD1556:BMPR-IA: V_m_ 3.06 Å^3^/Da or 60% solvent content; 4 AbD1556:BMPR-IA: V_m_ 2.29 Å^3^/Da or 46% solvent content). To solve this ambiguity, a native Patterson map analysis and a selfrotation calculation were performed using the software Phenix xtriage and GLRF providing evidence for the presence of four AbD1556:BMPR-IA complexes per asymmetric unit. The structure was then solved by molecular replacement using the software Phaser and the structures of BMPR-IA_EC_ (from the complex BMP-2:BMPR-IA, PDB entry 1REW) and of the Fab with high affinity for the tetanus toxoid (PDB entry 1AQK). Using a stepwise search procedure in the software Phaser all four AbD1556:BMPR-IA complexes could be identified in the asymmetric unit of the crystal. The structure was then refined in an iterative procedure via refinement using the software Refmac 5.02 [22] and manually rebuilding using Quanta2006 (Accelrys). For refinement non-crystallographic symmetric (NCS) was employed for the four AbD1556:BMPR-IA_EC_ complexes in the asymmetric unit with the light and heavy chains of the Fab and BMPR-IA forming three individual NCS groups. All three NCS groups were then restrained with tight positional restraints throughout the refinement in Refmac 5.02, but the heavy chain of AbD1556 molecule 1 (chain H) was excluded from NCS refinement as the strands β6 and β7 of the constant heavy (C_H_) domains were shifted by about 1.4 to 1.8 Å compared to the other three C_H_ domains due to differences in the crystal lattice contacts (see **Fig. 1c**).

To account for anisotropy in the diffraction data 20 TLS groups were employed in the refinement, using 1 TLS group for each constant and variable region of the light and heavy chain of the Fab as well as for BMPR-IA. In the final round of model building a F_obs_-F_calc_ difference electron density map was used to identify solvent molecules. The final model exhibits an R-factor of 0.234 (R_free_ 0.280) and consists of 15323 protein atoms and 27 water molecules. In all four complexes in the asymmetric unit residues Pro34 to Val118 of BMPR-IA as well as residues Ile2 to Thr211 of the light chain of AbD1556 could be modelled, for the 33 N-terminal and the 11 C-terminal residues of BMPR-IA no traceable electron density was observed. Similarly, Asp1 and residues Glu212 to Ala213 of the AbD1556 light chain could not be identified in the electron density maps. For the heavy chain of the four AbD1556 moieties electron density allowed tracing of residues Gln1 to Lys221 only for one heavy chain (chain J), whereas several residues in the loops of the constant regions of the other three AbD1556 heavy chain could not be modelled (chain H: Lys136 to Thr142, Ser194 to Gln199, Lys221; chain I: Lys136 to Gly140, Lys221; chain J: Lys136 to Gly141). Residues Ser222 to Phe224 and the peptide sequence of the additional Strep-Tag at the C-terminus of the AbD1556 constant region heavy chain could not be traced in the electron density in any of the four AbD1556 moieties. Regions immediately ahead of or behind stretches of missing residues usually exhibit high temperature factors (B-factors ≥120) indicating dynamic disorder (**Fig. S1**). Conformingly, the affected loop areas have little or no crystal lattice contacts that could stabilize a defined loop conformation (**Fig. S2**). Further statistics for data acquisition and structure refinement are compiled in **Table 1**.

**Table 1:** Data acquisition and structure refinement.

Crystals and data processing	AbD1556:BMPR-IA_EC_ complex
Beamline	home source
Wave length	1.5418 Å
Space group	P2_1_
Unit cell	a = 89.32, b = 129.25 Å, c = 100.24 Å
	α = γ = 90°, β = 92.27°
Resolution	30.4 - 2.70 Å
	(2.80 - 2.70 Å)^a)^
Number of reflections collected	155123 (15796)
Number of unique reflections	59539 (6237)
Completeness	95.3 (97.8) %
Multiplicity	2.6 (2.6)
Rsym for all reflections	8.0 (34.1) %
<Intensity/σ>	8.2 (2.7)
χ^2^	0.98 (1.19)
Refinement statistics	
Rcryst	23.4 (32.0) %
Rfree (test set 5%)	28.0 (33.8) %
NCS groups^b)^	3 (heavy chain, light chain, receptor)
	heavy chain, residues 1–221
	light chain, residues 2–220
	BMPR-IA, residues 34–117
TLS groups	20 (1 for each C_H_, C_L_, V_H_, V_L_ and BMPR-IA)
r.m.s. deviation	
Bonds	0.013 Å
Angles	1.624°
Torsion period 1	7.650°
Torsion period 2	38.169°
Torsion period 3	21.787°
Torsion period 4	22.560°
NCS_heavy chain_	0.07 Å
NCS_light chain_	0.10 Å
NCS_BMPR-IA_	0.07 Å
Average *B*-Factor^c)^	64.1 Å^2^ (min. 36.9 Å^2^; max. 158.1 Å^2^)
Coordinate error (based on Rfree)	0.41 Å
Procheck analysis^d)^	
most favored region	85.7% (1466)
additional allowed region	13.9% (237)
generously allowed region	0.5% (8)
disallowed region	0.0% (0)

^a)^ Statistical analysis for the highest resolution shell is shown in parentheses.

^b)^ Non-crystallographic symmetry restraints were applied between the four complexes in the asymmetric unit. One NCS group was defined for the heavy chain, the light chain and the receptor ectodomain of BMPR-IA and restrained to the respective molecules in the other three AbD1556:BMPR-IA complexes except for the heavy chain of AbD1556 molecule 1. As strict NCS was not applicable (see also **Fig. 1C–F**) tight positional NCS restraints were used throughout the refinement using Refmac 5.02.

^c)^ The minimal and maximal B-factor for the backbone atoms are indicated, for a comparison of the B-factor distribution between all four AbD1556:BMPR-IA complexes see **Fig. S1**.

^d)^ The numbers in parentheses indicate the absolute number of residues present in the respective area of the Ramachandran plot.

### Surface plasmon resonance

Interaction analysis was performed on a Biacore 2000 system (GE Healthcare, Biacore). All measurements were performed at room temperature. BMP-2 and the antibody proteins were biotinylated using LC-NHS-biotin (Pierce) in 2fold molar excess. Biotinylated proteins were immobilized on flow cells 2, 3 and 4 of a streptavidin-coated CM5 sensorchip (GE Healthcare). Binding of wildtype and mutant BMPR-IA_EC_ was measured using HBS_500_ buffer (10 mM HEPES, 500 mM NaCl pH 7.4, 3.4 mM EDTA, 0.005% surfactant P20) at a flow rate of 10 µl min^−1^. Between measurements the biosensor surfaces were regenerated by perfusing with 4 M magnesiumchloride for 2 min. Sensorgrams were recorded at analyte concentrations of 1000, 500, 250, 125, and 62.5 nM. All measurements were corrected for non-specific interactions and bulk face effects by subtracting a control sensorgram recorded for flow cell 1 (blank immobilization). Data were evaluated employing a simple 1∶1 Langmuir-type interaction model and using the software BIAevaluation version 2.0. Apparent binding constants (K_D_) were obtained from the dose dependency of equilibrium binding and/or from the kinetic rate constants for complex formation (*k*_on_) and dissociation (*k*_off_) respectively. The mean standard deviations for all K_D_ values were <50%.

### Biological activity of Fab proteins in cell line C2C12

The mouse myoblast cell line C2C12 (ATCC, No. CRL-1772) was cultured in DMEM medium containing 10% fetal calf serum (FCS), and antibiotics (100 U ml^−1^ penicillin G and 100 µg ml^−1^ streptomycin). For alkaline phosphatase (ALP) expression assays, the cells were incubated for 72 h in medium containing 2% FCS and supplemented with BMP-2 in 96-well microplates [23]. After cell lysis, ALP activity was measured by *p*-nitrophenylphosphate conversion using an ELISA reader at 405 nm. To assess the effect of the antibodies on cell signaling, Fab proteins and BMP-2 were added simultaneously. For such inhibition assays the BMP was added at the concentration required for half-maximal response (EC_50_), which was determined in a standard ALP-assay before. All experiments were performed in duplicate and the data presented were obtained from at least two independent experiments (**Fig. S3**).

## Results

### Crystallization of the BMP receptor-Fab complex BMPR-IA_EC_/AbD1556

To determine the structural plasticity of the BMP receptor IA binding epitope for binding to BMP-2 we prepared Fab proteins raised against the extracellular domain of BMPR-IA (obtained in collaboration with AbD-Serotech). The antibody fragments were generated by phage display using biotinylated BMPR-IA in a so-called solution panning [19] and employing the HuCal Fab library of AbD-Serotech [17], [18]. The binding properties of eight different Fabs directed against BMPR-IA_EC_ were analyzed using surface plasmon resonance and with respect to their BMP-2 neutralizing activity, using an alkaline phosphatase (ALP) expression assay. For two Fab proteins, AbD1556 and AbD1564, a BMP-2 neutralizing effect was observed, suggesting that these two antibody proteins bind BMPR-IA via the same or an overlapping epitope with BMP-2. Complexes of both Fab proteins with BMPR-IA_EC_ were prepared and subjected to crystallization trials. Although crystals of both complexes could be obtained, crystals of the complex AbD1564:BMPR-IA_EC_ diffracted only to low resolution and single crystals could not be obtained. In contrast, large crystals of the complex AbD1556:BMPR-IA_EC_ could be reproducibly grown, which also diffracted to high resolution. SDS-PAGE analysis of protein crystals confirmed that these crystals contain both proteins, the Fab AbD1556 and the BMPR-IA ectodomain.

A complete diffraction dataset of this Fab-BMP receptor complex was measured. The structure was determined by molecular replacement and using the structure of a Fab (PDB entry 1AQK, [24]), having high primary structure similarity as a search template. Since analysis of the unit cell content suggested the presence of up to four Fab:receptor complexes in the asymmetric unit, it was unclear whether all Fab fragments would have a BMPR-IA moiety bound. Using the software Phaser for the molecular replacement and an iterative search procedure, four Fab AbD1556:BMPR-IA_EC_ complexes were found in the asymmetric unit. The initial search using the full Fab fragment of a Fab directed against Tetanus toxoid, which shared the highest amino acid similarity with the Fab antibodies of AbD Serotec, failed, suggesting that the architecture of the Fab of AbD1556 in complex with BMPR-IA_EC_ differs from the template. A comparison of the final structure of AbD1556 (**Fig. 1A,B**) and 1AQK indeed shows that although the orientations of both constant regions of heavy (C_H_) and light (C_L_) chain as well as of both variable regions of constant and heavy chain are identical, the orientation of the variable region towards the constant region differs significantly in both Fab proteins, AbD1556 and the template Fab PDB entry 1AQK. Superposition of the variable domains of either the heavy (V_H_) or light (V_L_) chain shows that the constant regions reorient by 38° and 43°.

### Architecture of the Fab AbD1556:BMPR-IA complex and epitopes involved in binding

A comparison of the four complexes in the asymmetric unit suggests that all four of the Fab:BMPR-IA_EC_ complexes are almost identical, as a global fitting yields r.m.s. deviations of 0.6 to 0.7 Å for all Cα-atom positions. However, a detailed analysis reveals that the substructures, i.e. the Fab fragment or the receptor ectodomain, are identical within the accuracy of the acquired data. Backbone as well as side chain atoms of the four Fab fragments in the asymmetric unit superimpose almost perfectly with most pairings exhibiting an root-mean-square deviation (r.m.s.d.) of 0.6 Å or less, and superposition of the four BMPR-IA receptor ectodomains yields an r.m.s.d. of 0.16 Å or less. However, when the Fab molecules are structurally aligned a comparison of the bound BMPR-IA ectodomains shows that the receptor molecules shift slightly in the four complexes in the asymmetric unit (**Fig. 1C–F**). In the Fab:BMPR-IA_EC_ complex 1 of the asymmetric unit the BMPR-IA moiety is shifted by 0.8 to 1 Å towards the heavy chain compared to the three other Fab:receptor ectodomain complexes of the asymmetric unit. Both the Fab moiety as well as the receptor ectodomain moiety seem to act as rigid bodies, thus these differences show that a small reorientation of BMPR-IA within the binding epitope of the antibody protein is possible.

**Figure 2:**
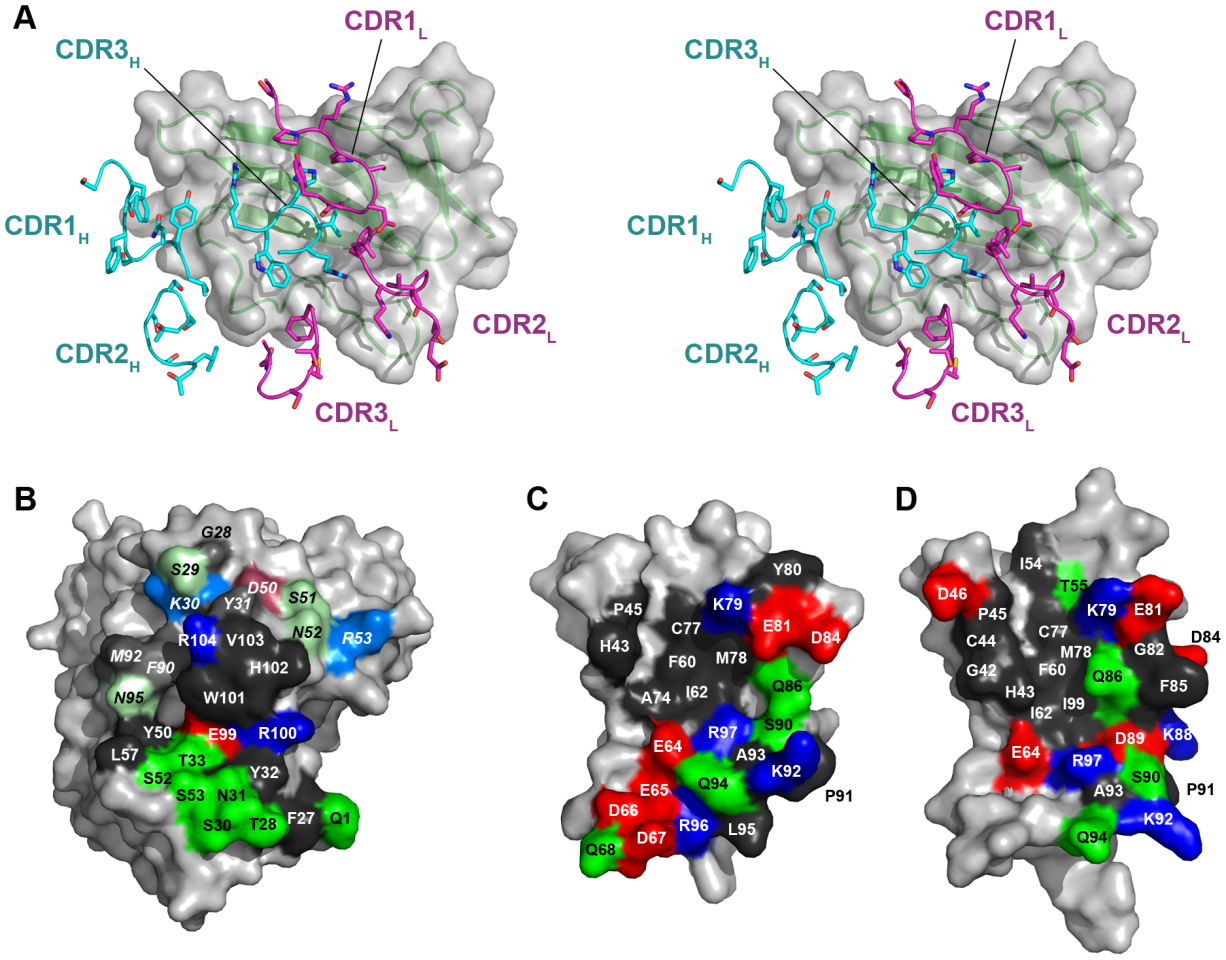
Binding epitopes of AbD1556 and BMPR-IA. (**A**) Stereo view of the binding epitope of BMPR-IA in the AbD1556:BMPR-IA complex. The positions of the six CDR binding loops are shown highlighting the central location of CDR3 of the heavy chain of AbD1556. Besides CDR3 of the heavy chain only the CDRs 1 and 2 of the light chain make significant contact with BMPR-IA. (**B,C**) Open book view of the binding epitopes of AbD1556 (B) and BMPR-IA_EC_ (C). The binding epitope is color-coded with hydrophobic amino acid residues (A, C, F, G, H, I, L, M, P, V, W, and Y) in dark gray, acidic residues in red (D, E), basic residues in blue (K, R) and polar but uncharged residues in green (N, Q, S, T). Residues in the epitope of AbD1556 originating from the light chain are indicated by brighter color and by italic labels. (**D**) The binding epitope of BMPR-IA for binding to BMP-2 (orientation of BMPR-IA in (C) and (D) are identical) showing that both epitopes are highly similar and overlap almost perfectly.

Upon complex formation about 1110 Å^2^ solvent-accessible surface area of the Fab fragment and 1230 Å^2^ of the BMP receptor ectodomain are buried. As expected from other antigen-antibody structures the complementary determining region (CDR) 3 of the heavy chain contributes, by far, the most to the buried surface area (570 Å^2^), as the CDR3 of the variable heavy chain domain (V_H_) is in the centre of the antibody-antigen interface (**Fig. S4**). In the light chain it is CDR1 and 2 that contribute significantly (165 and 95 Å^2^) to the antibody interface, whereas CDR3 does not seem to be much involved in the binding (buried surface area 59 Å^2^) due to the location of V_L_ CDR3 at the periphery of the BMPR-IA binding site. In total, the CDRs of the light chain contribute only 319 Å^2^ surface area to the interface, in contrast to the CDRs of the heavy chain, which all together deliver almost 800 Å^2^ of interface area. One possible explanation for the uneven contribution of the V_H_ and V_L_ segments to the interface is the location of BMPR-IA_EC_ on top of the antigen-binding site of the Fab AbD1556. The V_L_ domain is placed opposite of the β1β2-loop and the C-terminal end of β-strand 4 of BMPR-IA, whereas the V_H_ domain covers most of the three-stranded central β-sheet and a large part of the β4β5-loop of the receptor ectodomain (**Fig. 2A**).

In the case of BMPR-IA, the interfacial residues of the AbD1556:BMPR-IA_EC_ complex are almost evenly distributed over the slightly concave site of the three-stranded β-sheet, which consists of the β-strands β3, β4 and β5, but the Fab-binding epitope also involves several residues from the β1β2-loops as well as the β4β5-loops. A comparison with BMPR-IA_EC_ bound to BMP-2 (PDB entry 1REW) shows that the binding epitopes on BMPR-IA overlap heavily in both complexes (**Fig. 2B–D**); even the amount of interface area involved for BMPR-IA is quite similar (BMPR-IA_AbD1556_ 1230 Å^2^ vs. 1200 Å^2^ for BMPR-IA_BMP-2_). A closer inspection, however, also shows differences regarding to the extent that individual residues participate in the interface (**Fig. S4**). In the BMP-2:BMPR-IA_EC_ complex the β1β2-loop of the receptor ectodomain is more tightly packed into the interface as compared to the AbD1556:BMPR-IA_EC_ complex, whereas in the latter β-strand 3 and the C-terminal end of the β4β5-loop of BMPR-IA are most deeply buried.

**Figure 3:**
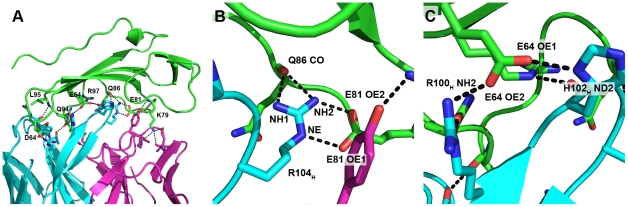
Hydrogen bond network in the AbD1556-BMPR-IA interface. (**A**) Overview of the hydrogen bonding between AbD1556 (shown in cyan and magenta for the heavy and light chain, respectively) and BMPR-IA_EC_ (green). Only residues involved in hyydrogen bonds across the interface are shown. (**B**) Zoom into the hydrogen bond cluster around Glu81 of BMPR-IA. (**C**) Magnification of the hydrogen bond cluster around Glu64.

Seventeen hydrogen bonds between the Fab and the receptor ectodomain document the rather polar character of the antibody-receptor interaction (**Table 2**, **Fig. 3A**). Most hydrogen bonds to the Fab involve the β4β5-loop segment of BMPR-IA (eight hydrogen bonds for residues Glu81 to Arg96), which also carries the main determinants for binding to BMP-2 [11]. This suggests that either amino acid composition or the conformation of this loop render this segment “sticky” for protein-protein interactions. Two residues in the Fab-receptor complex form hydrogen bond clusters or networks. Four hydrogen bonds emanate from the guanidinium group of Arg104 of the Fab heavy chain to the carboxylate group of BMPR-IA Glu81, forming a bi-dentate saltbridge, and two further hydrogen bonds are formed with the backbone carbonyl of BMPR-IA Gln86 (**Fig. 3B**). The large number of hydrogen bonds, together with the fact that these residues are rather deeply buried inside the interface, indicates that Arg104 of the Fab AbD1556 (and possibly Glu81 of BMPR-IA as well) might represent so-called hot spots of binding. The second independent hydrogen bond network is centred on BMPR-IA Glu64, whose side chain carboxylate group forms hydrogen bonds with the side chains of Arg100 and His102 of the heavy chain of Fab AbD1556 (**Fig. 3C**). Both latter side chains are pre-oriented in the interface either by an additional hydrogen bond, i.e. Fab Arg100 to Gln94 of BMPR-IA or by hydrophobic contacts, i.e. Fab His102 in contact to BMPR-IA Ile62 and Fab V_L_ Tyr48.

**Table 2:** Geometry of hydrogen bonds in the AbD1556 - BMPR-IA interface.

BMPR-IA_EC_	AbD1556	Distance Å	Angle NOC^a)^	Hydrogen bond^b)^
E64 (OE1)	H102_H_^c)^ (ND1)	2.38	151	SC-SC
E64 (OE2)	R100_H_ (NH1)	3.00	105	SC-SC
D67 (OD1)	Y32_H_ (OH)	3.06	109	SC-SC
D67 (OD2)	R98_H_ (NH2)	2.39	137	SC-SC
K79 (N)	Y31_L_ (OH)	2.70	117	MC-SC
K79 (NZ)	G28_L_ (O)	3.06	104	SC-MC
K79 (NZ)	K30_L_ (O)	2.79	100	SC-MC
K79 (NZ)	N65_L_ (OD1)	3.21	156	SC-SC
E81 (OE1)	R104_H_ (NE)	2.82	123	SC-SC
E81 (OE2)	R104_H_ (NH2)	2.46	121	SC-SC
Q86 (O)	R104_H_ (NH1)	3.08	91	MC-SC
Q86 (O)	R104_H_ (NH2)	2.96	96	MC-SC
K92 (O)	T33_H_ (OG1)	2.47	114	MC-SC
K92 (NZ)	N95_L_ (O)	2.81	124	SC-MC
Q94 (OE1)	R100_H_ (NE)	2.78	99	SC-SC
L95 (N)	N31_H_ (O)	3.03	111	MC-MC
R97 (NE)	H102_H_ (O)	2.71	132	SC-MC
mean value		2.79	115	
S.D.		0.26	19	

The numbers in parentheses are the distances between donor and acceptor atoms and N-O-C angles in the AbD1556:BMPR-IA interface of complex 1 in the asymmetric unit.

^a)^ N, O, C are the donor-acceptor atoms; from general statistics [48] this angle is 149°±15° for MC–MC hydrogen bonds and 129°±18° for SC-MC and SC-SC hydrogen bonds;

^b)^ MC (main chain) and SC (side chain) donor/acceptor atoms;

^c)^ H and L denote heavy and light chain of the Fab AbD1556, respectively.

### The structures of BMPR-IA in complex with the Fab AbD1556 and BMP-2 differ

Due to the overlapping epitopes of Fab AbD1556 and BMP-2 on BMPR-IA one can think of the Fab AbD1556 binding epitope as similar to an anti-idiotypic molecule mimicking BMP-2. It is thus an interesting question whether the conformations of BMPR-IA_EC_ bound to either the Fab AbD1556 or BMP-2 are identical, especially since our NMR-studies of isolated BMPR-IA_EC_ have shown that the majority of the BMP-2 binding epitope of BMPR-IA is unfolded before binding [15]. The large conformational change upon binding to BMP-2 is mostly apparent from the formation of a short α-helix segment (helix α1) comprising residues Gly82 to Lys88 in the β4β5-loop. This α-helix, which contains the binding hot spots for the interaction of BMPR-IA with BMP-2 [11], is absent in the free form of BMPR-IA, but formed spontaneously in NMR-titration experiments using the helix-inducing agent trifluoroethanol [15]. It was thus suggested that the helical element would be in a so-called *status nascendi* thereby forming instantaneously upon changes in the environment without the need of a BMP-2 “casting mold”.

**Figure 4:**
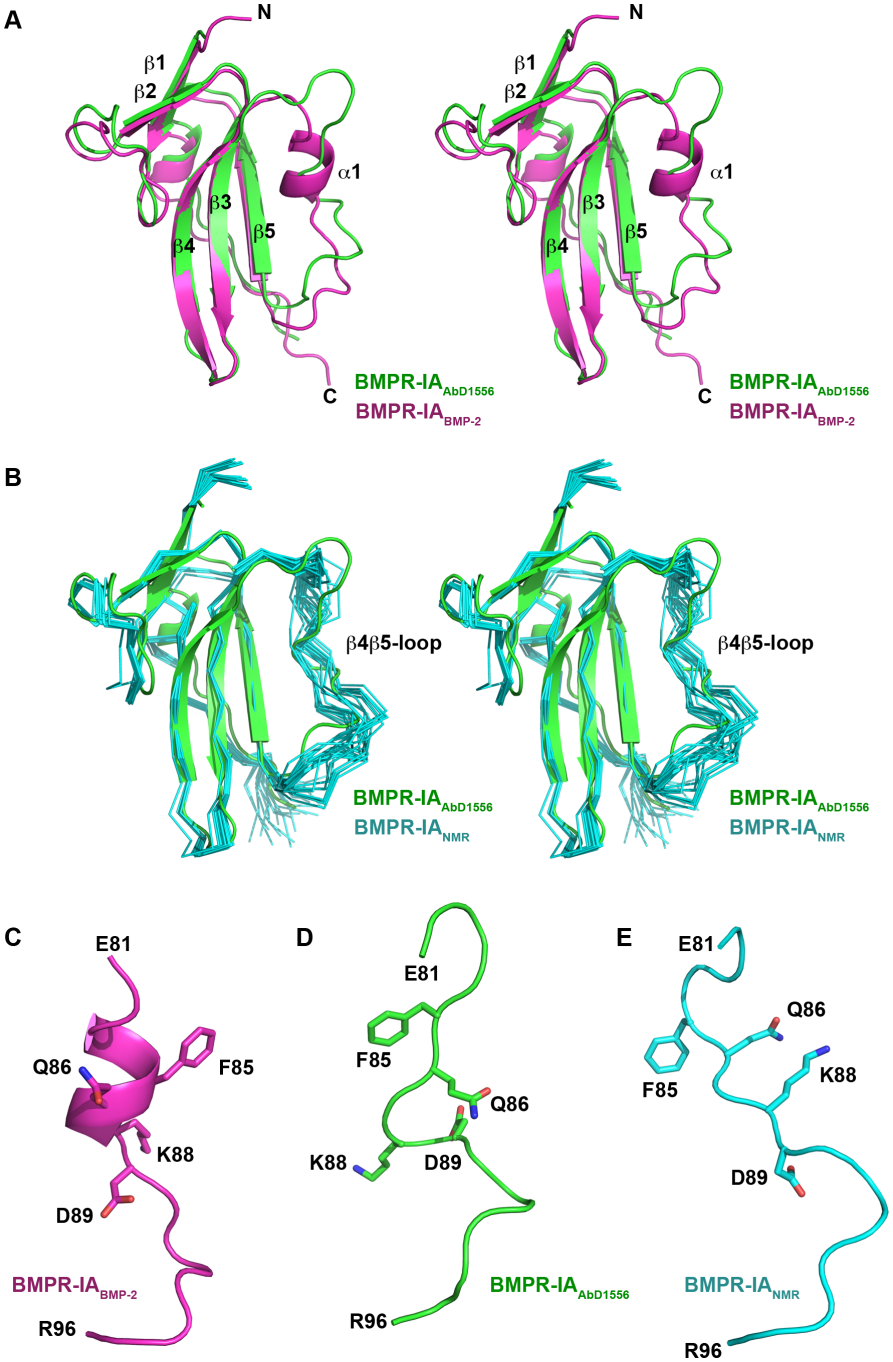
Comparison of the bound and unbound forms of BMPR-IA. (**A**) Structural superposition (Stereoview) of BMPR-IA_EC_ bound to AbD1556 (green) and bound to BMP-2 (magenta, PDB entry 1REW). The comparison shows that the short α-helix 1 that is observed in the complex BMP-2:BMPR-IA is absent when BMPR-IA is bound to AbD1556. The β4β5-loop adopts two different conformations in the two BMPR-IA structures. (**B**) (Stereoview) Structural alignment of the NMR-ensemble of BMPR-IA_EC_ (Cα-trace of 21 structures in cyan, PDB entry 2K3G) and BMPR-IA_EC_ bound to AbD1556. The β4β5-loop of BMPR-IA bound to AbD1556 has only partial similarity to the structures of the NMR ensemble. (**C–E**) Comparison of the β4β5-loop of BMPR-IA bound to BMP-2 (C), bound to AbD1556 (D) and for a representative conformer of the free form (E). The side chains of residues Phe85, Gln86, Lys88 and Asp89 that play an important role in binding to BMP-2 or AbD1556 show very different orientations in all three structure (forms).

However, a comparison of BMPR-IA bound to Fab AbD1556 and BMPR-IA bound to BMP-2 (PDB entry 1REW) clearly shows that the helix α1 is also absent in the former (**Fig. 4A**). This segment of BMPR-IA adopts a rather irregular conformation with short extended stretches, i.e. Asp84 to Cys87 and Asp89 to Pro91. Interestingly, despite the difference in the conformations of the β4β5-loop in the NMR structure (PDB entry 2K3G) and the complex structure when bound to AbD1556, all other secondary structure elements, e.g. the three-stranded β-sheet or the N-terminal β-strands β1 and β2 together with the β1β2-loop, overlap almost perfectly (**Fig. 4A**). Since the conformational rearrangement in BMPR-IA could principally follow either an induced fit or a selection of conformer mechanism, we also compared the structure of BMPR-IA bound to Fab AbD1556 also with all the individual structures of the NMR ensemble (**Fig. 4B**). However, this comparison shows that although the loop is also irregular in the NMR structure ensemble, there is no conformer in the ensemble, which is identical to BMPR-IA bound to AbD1556. Moreover, the conformation of the BMPR-IA β4β5-loop in its AbD1556 bound form seems to be a mixture of the conformations seen in the NMR ensemble and the BMP-2 bound form. Residues Ser90 to Ala93 overlap with the conformer ensemble present in the NMR structures, while Asp89 to Gln86 show a helical twist similar to the helical end in BMPR-IA when bound to BMP-2. Only the N-terminal part of the β4β5-loop, i.e. residues Gly82 to Phe85 exhibits a conformation which is neither present in the NMR ensemble nor the BMP-2 bound form (**Fig. 4C**). This observation suggests that an induced fit mechanism, where the flexible, disordered (in its free form) β4β5-loop BMPR-IA adopts a conformation imposed by the binding partner. In BMP-2 the helix is formed because the highly concave site of the wrist epitope forms a 120° spanning binding site, which is usually seen in structural arrangements of amphipathic helix/helices (e.g. hydrophobic cores in helical bundles). In contrast, the binding site of the Fab is rather flat, thus the interaction to stabilize the helical element is not sufficient and the β4β5-loop adopts a more extended, helical-free structure.

### Main binding determinants of BMPR-IA for AbD1556 and BMP-2 are identical

As mentioned above, the structural epitopes of BMPR-IA buried upon binding to the Fab AbD1556 or to BMP-2 overlap heavily, and an almost identical set of residues in the receptor ectodomain facilitates binding to both ligands. Therefore, a mutational analysis of BMPR-IA was performed in order to see whether the functional epitopes, i.e. the main binding determinants for the interaction with both binding partners, are similar. If the Fab AbD1556 would function as a BMP-2 mimic, one would expect that the same residues play similar roles in the recognition and binding of both partners. However, the different number of hydrogen bonds between BMPR-IA and AbD1556 compared to the complex of BMPR-IA and BMP-2 as well as the different residues involved on BMPR-IA suggests that the functional epitopes of BMPR-IA for AbD1556 and BMP-2 are markedly different. We therefore mutated 13 receptor residues in the centre of the contact surface. In most cases a mutation to alanine was done, but at three positions we also substituted these residues for Asp, Arg, or Pro (**Table 3**). The resulting mutant proteins were purified, and their binding affinities were studied by surface plasmon resonance. In order to measure comparable binding affinities without avidity effects resulting from different oligomeric states (BMP-2 as homodimer will bind to two receptor molecules simultaneously if used as analyte) we immobilized both the monovalent Fabs AbD1556 and AbD1564 (another neutralizing anti-BMPR-IA Fab) as well as BMP-2 in different flow cells of one biosensor chip. Each BMPR-IA variant protein was then perfused as analyte over all three ligands in parallel, which allows for direct comparison of the binding data (**Tables 3** **and** **4**).

**Table 3:** Mutational analysis of the BMPR-IA binding interface^a)^.

	BMP-2	AbD1556	AbD1564
	rel. app. K_D_		
BMPR-IA WT	1.0±0.1	1.0±0.1	1.0±0.2
	(29.7±2.8)	(18.6±1.2)	(55.7±6.6)
[P34R]	1.7	1.2	0.9
[Y39D]^b)^	16.4	23.5	n.b.
[F60A]^b)^	n.b.^c)^	n.b.	n.b.
[I62A]	**42.4**	**29.1**	1.8
[E64A]	1.3	2.3	0.5
[D67A]	1.0	1.7	***n.b.***
[M78A]^b)^	n.b.	n.b.	3.8
[K79A]	0.4	0.7	***n.b.***
[E81A]	2.3	2.3	0.5
[F85A]	***n.b.***	***n.b.***	0.8
[Q86A]	**17.3**	**27.1**	0.8
[K92A]	1.8	1.7	***n.b.***
[Q94A]	1.2	1.2	**19.5**
[Q94P]	3.8	2.9	5.3
[R97A]	**34.2**	**13.4**	***n.b.***
[I99A]	**25.1**	**19.8**	4.1

^a)^ Contribution of the mutated residue to the overall binding energy is indicated by bold, italic letters (contribution very strong) or only boldface letters (contribution strong).

^b)^ Both BMPR-IA variant proteins could only be produced in non-homogenous form indicating variants that have lost the native BMPR-IA fold, thus these variants were excluded from analysis.

^c)^ n.b.: no binding. SPR data could not be fitted due to too low binding signal, however, from the analyte concentration used in the analysis, a lower limit for the binding affinity can be estimated to K_D_ ≥5 µM.

**Table 4:** Kinetics of the interaction of BMPR-IA with Fabs and BMP-2.

	BMPR-IA_EC_ WT		
	*k*_off_ [×10^−3^ s^−1^]	*k*_on_ [×10^4^ M^−1^s^−1^]	*K*_D_ (nM)
BMP-2	1.9	7.2	27
AbD1556	1.7	8.8	20
AbD1564	7.1	15	49

**Figure 5:**
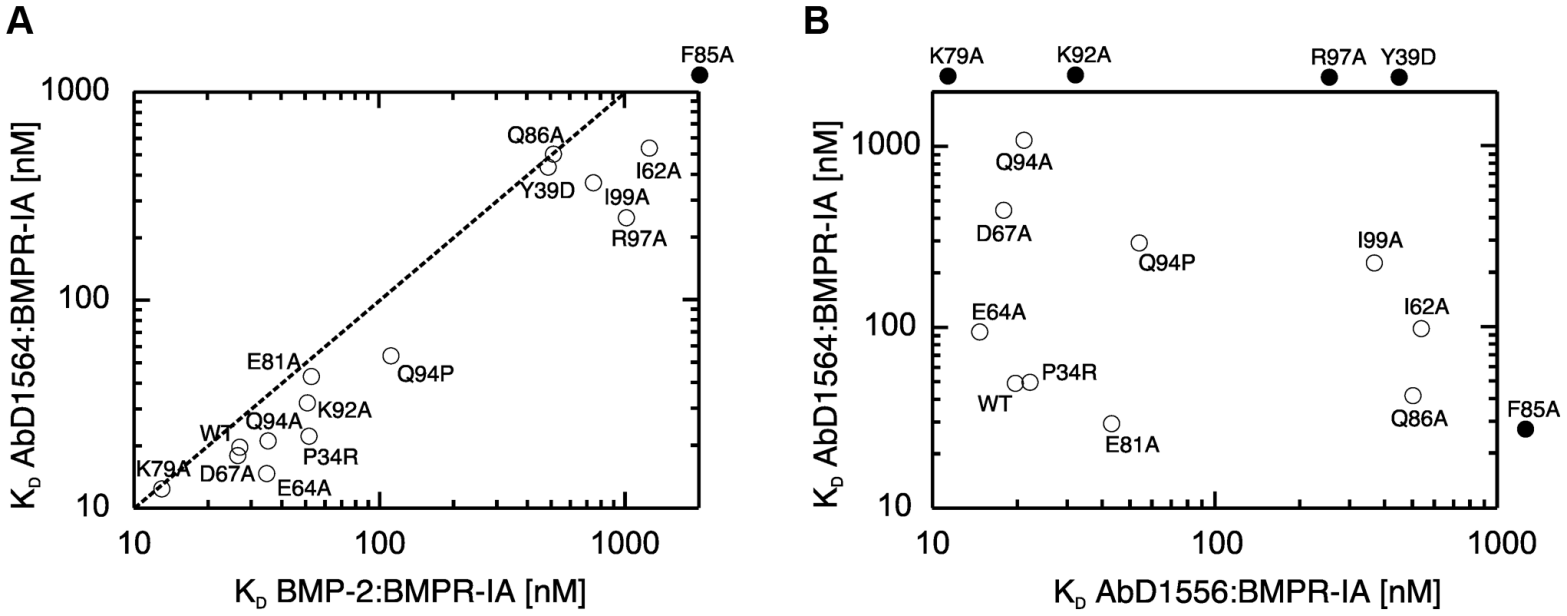
Functional epitopes of AbD1156, AbD1564 and BMP-2. Mutational analysis of the BMPR-IA binding epitope and *in vitro* interaction analysis shows that the contribution of residues within the epitope to the binding energy correlate for binding to AbD1556 and BMP-2 (**A**), whereas the two Fab AbD1556 and AbD1564 clearly bind to BMPR-IA via a different mechanism (**B**).

It was very surprising to see that the functional epitopes of BMPR-IA for BMP-2 and AbD1556 are highly similar despite the structural differences in the epitopes of BMPR-IA for binding either to BMP-2 or AbD1556. The affinities of the different receptor mutants for BMP-2 and AbD1556 investigated here were clearly correlated (see apparent K_D_'s in **Fig. 5A**). One set of BMPR-IA mutants exhibits affinities for AbD1556 and BMP-2 similar to the wild type BMPR-IA. Interestingly, this set comprises almost all of the polar residues, i.e. Glu64, Asp67, Lys79, Glu81, Lys92, and Gln94, within the Fab-binding epitope of BMPR-IA, which form 11 out of 15 intermolecular hydrogen bonds in the Fab-receptor interface. Thus polar interactions, despite their large number, seemingly do not contribute significantly to the binding energy. This unexpected finding might be explained by two factors. Firstly, many of these hydrogen bonds originate from side chains of BMPR-IA residues that are located at the periphery of the interface and, thus, might be solvent-accessible. Without shielding from the solvent, the hydrogen bonds to the protein partner compete with hydrogen bonds with water and the energetic benefit for the protein-protein interaction will hence be low. Secondly, eight of the eleven addressed hydrogen bonds are of the type “side chain to side chain”, meaning that the entropy cost from decreasing the conformational flexibility of both side chains involved in the intermolecular hydrogen bond might just be barely paid off by the bond formed. A similar observation is reported for the BMPR-IA:BMP-2 interaction [11], where only two of the ten intermolecular hydrogen bonds are significant for the ligand-receptor complex formation. Another set of BMPR-IA mutants (Ile62, Gln86, Arg97, and Ile99) showed affinities for Fab AbD1556 (or for BMP-2) that are 10- to 30-fold (for the interaction with BMP-2: 20- to 40-fold) lower compared to wild type BMPR-IA. A third group of BMPR-IA mutants exhibited no measurable affinity for AbD1556 or BMP-2. Three of those mutants (Y23D, F60A and M78A) have likely lost the native fold of BMPR-IA, because these variant proteins could only be obtained in non-homogeneous form and with very low yield. The BMPR-IA variant F85A, however, seems structurally intact and binds to neither AbD1556 nor BMP-2.

**Figure 6:**
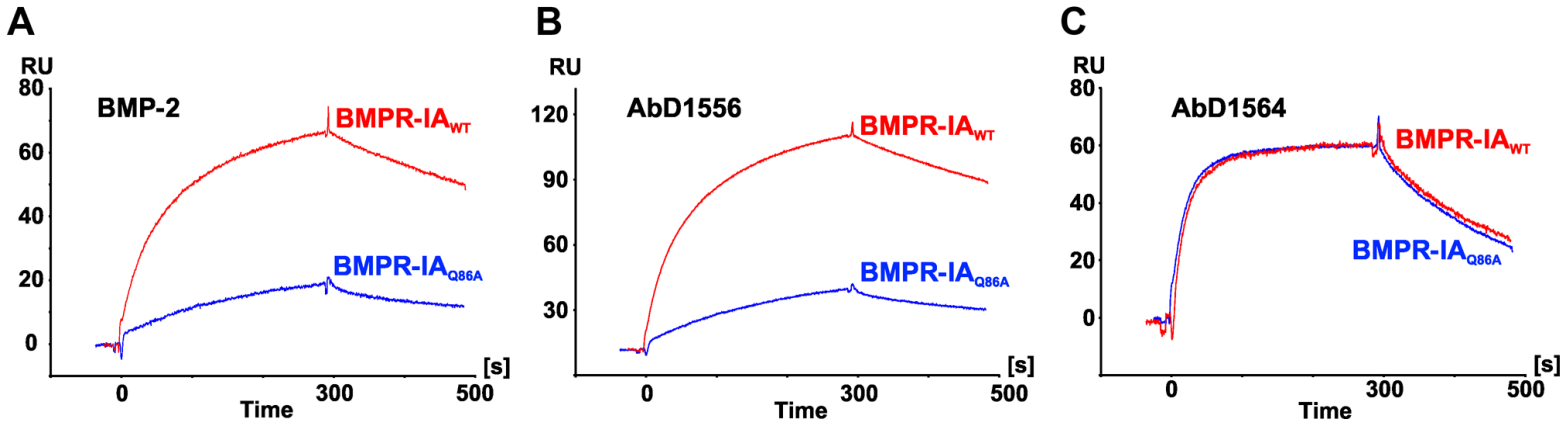
SPR analysis of BMPR-IA binding to BMP-2 and Fab proteins. Sensorgram of the interaction of wildtype BMPR-IA (red) and the BMPR-IA variant Q86A (blue) with BMP-2 (**A**), AbD1556 (**B**) and AbD1564 (**C**). The sensorgrams were recorded using BMPR-IA or the variant BMPR-IA [Q86A] as analytes at a concentration of 250 nM. At time point 0 the analytes were added (association phase), at time point 300 s only buffer was perfused allowing to monitor the dissociation of the receptor protein from the biosensor.

The same set of BMPR-IA variants was also tested for binding to another Fab, AbD1564, which due to its BMP-2 neutralizing properties must have a BMPR-IA epitope (at least partially) overlapping with that of BMP-2. Unfortunately, the lack of structural data for AbD1564 does not allow for a direct comparison of the structural epitopes. However, the comparison of both functional epitopes of BMPR-IA for binding to either AbD1556 or AbD1564 shows that residues of BMPR-IA contribute vastly different to the binding of the two Fab proteins (**Fig. 5B** **,** **Table 3**). Whereas the mutation of a contact residue in BMPR-IA results in a highly similar change in binding affinity for BMP-2 and AbD1556 (as evident from the correlation factor K_D_(BMPR-IA:BMP-2)/K_D_(BMPR-IA:AbD1556) being located on or close to the diagonal in **Fig. 5A**), no correlation exists when the contribution of individual BMPR-IA residues to binding energy is compared for the interaction of BMPR-IA with AbD1556 and AbD1564 (**Fig. 5B**). The results are surprising since the functional epitopes of BMPR-IA for binding to BMP-2 and AbD1556 seem identical despite the fact that the structures of BMPR-IA bound to either BMP-2 or AbD1556 differ significantly. Specifically, the exchange of the BMPR-IA residues Phe85 and Gln86 to alanine renders the binding to AbD1556 similar as to BMP-2, although neither side chain directly contact the Fab surface. A closer inspection of the kinetic rate constants of the AbD1556-BMPR-IA mutant interaction shows that in the case of BMPR-IA Q86A only the association rate is affected (**Table 5**, **Fig. 6**). Since the interaction of Fab AbD1564 with BMPR-IA is not affected by the mutation Q86A (**Fig. 6C**) a global misfolding altering the binding properties of the BMPR-IA variant can be ruled out. Analysis of the association and dissociation rates strongly indicates that the binding mechanism of BMPR-IA to AbD1556 and BMP-2 follows a selection-fit (or selection of conformation) mechanism (**Fig. 6**). Thus the strong influence of the mutations F85A and Q86A in BMPR-IA on binding to AbD1556 is likely due to a shift in the conformer population eliminating or reducing the fraction of molecules adopting the conformation required for binding to AbD1556. Supportive of such a selected fit mechanism are the rather slow association rates, e.g. 7 to 9×10^4^ M^−1^s^−1^ for the BMPR-IA interaction with AbD1556 or BMP-2, whereas the association with the second Fab AbD1564 occurs twice as fast (1.5×10^5^ M^−1^s^−1^) (**Table 4**) [25]. Furthermore, the observed effect that mutations at the periphery of the interface influence the binding affinity, even though the mutated residues are not directly contacting the binding partner, is in concordance with the theory that those residues affect the population equilibrium of the different conformers and thereby indirectly affect binding [25]. In summary, the comparative structure-/function analysis of the BMPR-IA interaction with the Fab AbD1556 and its ligand BMP-2 suggests that the location of the binding determinant, rather than its chemical nature, is the most important factor contributing to the overall binding energy.

**Table 5:** Relative kinetic rate constants of the interaction of BMPR-IA and variants thereof with BMP-2, AbD1556 and AbD1564^a)^.

	BMP-2		AbD1556		AbD1564	
	rel. *k*_off_	rel. *k*_on_	rel. *k*_off_	rel. *k*_on_	rel. *k*_off_	rel. *k*_on_
BMPR-IA	1.0	1.0	1.0	1.0	1.0	1.0
[P34R]	1.0	0.6	0.8	0.8	0.7	0.7
[I62A]	**24.0**	1.5	**17.5**	0.7	1.1	0.6
[E64A]	0.9	0.8	2.7	1.3	0.7	1.4
[D67A]	0.8	0.8	1.8	1.1	-^b)^	-^b)^
[K79A]	0.4	1.0	0.6	0.9	-^b)^	-^b)^
[E81A]	2.5	1.4	2.7	1.3	0.7	1.4
[F85A]	-^b)^	-^b)^	-^b)^	-^b)^	1.1	1.4
[Q86A]	2.3	*0.1*	2.4	*0.1*	0.7	0.9
[K92A]	1.8	1.0	1.8	1.1	-^b)^	-^b)^
[Q94A]	1.2	1.0	1.2	1.1	-^b)^	-^b)^
[Q94P]	**5.8**	1.7	**4.8**	1.7	**15.7**	3.1
[R97A]	-^b)^	-^b)^	**26.3**	2.5	-^b)^	-^b)^
[I99A]	**6.0**	*0.2*	**6.4**	0.4	1.3	0.3

^a)^ Only BMPR-IA variants for which the native BMPR-IA fold can be assumed were included in the analysis. Relative rate constants are shown, absolute rate constants are given in Table 4a. Whether the mutation of the respective amino acid residue to alanine affects the dissociation is marked by boldface letters, if the association kinetics is affected rate constants are shown in italic letters. Changes larger than 3fold are considered significant.

^b)^ Due to the very fast interaction kinetics observed for those BMPR-IA variant proteins, analysis of the rate constants was impossible, binding equilibrium constants were thus obtained from analysis of the dose-dependency of equilibrium binding.

## Discussion

In this study, we present the structure of the BMP type I receptor BMPR-IA bound to the antibody fragment AbD1556, which is an efficient inhibitor for BMP mediated signalling. To date, more than 1300 structures for antibodies, fragments thereof (F_v_, Fab, sF_v_, camelid antibodies) or complexes with antigens or small molecule haptens are available in the RCSB structure databank, demonstrating the enormous interest in this protein class. Their versatile binding properties have been structurally and functionally studied since the early 90′s providing a wealth of data on how antibodies in principle recognize and bind their target molecules [26], [27]. The Fab AbD1556 binding to the BMP receptor IA is not an exception to the rules that have been derived from other antibody structures. Thus the binding epitope of Fab AbD1556 has the expected size for binding to a rather large protein antigen, the binding site is dominated by the variable region of the heavy chain, particularly by CDR3_H_ (residues Glu99 to Phe107, Kabat numbering 95 to 100C), which lies in the centre of the contact. The loop conformations of the CDRs L1, L2, H1 and H2 follow the classification of Chothia, exhibiting the canonical structure classes 4, 1, 1 and 3, respectively [28], [29]. The CDR3 loop of the light chain is unusually long and seems not to obey any of the known classes. As observed in other antibody-antigen structures, the number of serine residues is indeed increased in the CDR loops. Due to the rather large size of the antigen BMPR-IA, the binding epitope of the Fab is quite flat maximizing the contact between the CDR loops and the antigen. It therefore seems like that the antigen binding mechanism of the Fab AbD1556 does not differ very much from those observed in other antibody-antigen interactions.

Thus the far more interesting aspect lies in the structure of the antigen, the BMP receptor type IA. The structure, when bound to the neutralizing Fab AbD1556, reveals an epitope on BMPR-IA highly overlapping structurally with that of BMPR-IA when bound to its natural ligand BMP-2. Comparing the binding sites on BMP-2 and AbD1556, which might be considered as an anti-idiotypic BMP-2 potentially exhibiting a similar binding surface as BMP-2, shows that there is very limited similarity in surface shape (**Fig. S5**). In the BMPR-IA:BMP-2 complex, BMPR-IA is buried in a deep, quite concave cleft on BMP-2, whereas in the Fab:BMPR-IA complex the binding site on the Fab is very flat. On the other hand, in both sites the epitope center is dominated almost exclusively by hydrophobic amino acids, which are surrounded by a ring of polar and/or charged residues, suggesting that the surface chemistry involved are similar despite the structural differences.

A previous NMR structure analysis of BMPR-IA illustrated that the ligand-binding epitope of BMPR-IA is largely unordered in its unbound form and subject to a large conformational rearrangement upon BMP-2 binding [15]. The main changes are located in the long loop between the β-strands 4 and 5 of the BMPR-IA ectodomain. In its BMP-2 bound form this loop adopts a rigid extended conformation with a short 1.5turn helix comprised of residues Ser83 to Lys88 [11], [12]. NMR titration studies using the helix-inducing agent trifluoroethanol point towards an induced fit mechanism, which seems to lead to a spontaneous helix induction upon changes in the environment [15]. However, in the structure of BMPR-IA bound to the Fab AbD1556 the β4β5-loop adopts a rather different conformation, which resembles a mixture of the conformation(s) found in the NMR structure ensemble and the BMP-2 bound form. Thus the helix formation does not precede complex formation and whether a helix is formed in the BMPR-IA β4β5-loop depends on the nature of the binding partner. This structural plasticity of the BMP type I receptor epitope, which allows BMPR-IA to adapt to different binding partners without deteriorating binding affinities to either one of the partners is possibly a key mechanism by which proteins can achieve binding to more than one interaction partner utilizing the same binding site. The so-called binding promiscuity has become an increasingly observed phenomenon in numerous protein superfamilies rather than an exotic exception (for a limited number of examples, see [7], [30], [31], [32], [33], [34]). For some protein families, e.g. antibodies, T-cell receptors and other “receptors” of the immune system, binding to numerous (or even an unlimited number of) other proteins is a generally accepted phenomenon, but it has still been assumed that a particular antibody would still recognize only a single antigen. An antibody binding to different antigens, i.e. showing cross-reactivity, was considered rare and its promiscuous binding due to structural similarities between the cognate antigens. A promiscuous protein is defined as protein with a defined sequence that can bind various partners (but still exhibits high specificity for this particular group of partners) without utilizing different epitopes, making promiscuity an incomprehensible mystery in protein-protein recognition. In its most extreme form, so-called hub proteins at central positions of interaction networks were postulated, which supposedly bind to tens or even hundreds of partners [35], [36]. Although this finding possibly might explain the numerical discrepancy between the rather small number of genes and the complexity of the signaling network in complex organisms such as humans [37], it raises questions about how a single hub protein can interact with so many different partners [38]. Several solutions to this logical obstacle have been proposed. Many hub proteins exhibit a modular architecture in which several different protein interaction domains, that typically bind linear epitopes, are shuffled together [39]. Depending on post-translational modifications or splicing, these hub proteins can form different interaction complexes thereby enhancing the complexity. The discovery of intrinsically disordered proteins has added another powerful example how protein-protein interactions (networks) can be extended [40]. The often under-appreciated fact that proteins are inherently flexible, from side chain to backbone level, allows proteins to adapt to different binding sites [41], [42]. Although neither BMPR-IA nor its BMP ligands are classical hub proteins, the manifold interactions of BMPR-IA and other BMP type I receptors with various BMPs as well as the interaction of these BMPs with other receptors, co-receptors or modulator proteins show similarities to hub proteins in these interaction networks. Neither the ligand nor the receptor are modular proteins, but modularity of binding epitopes has been described to allow for promiscuity [43]. Cooperativity from using two (or several) discontinuous binding elements as observed in the interaction of the von Willebrand domain VWC1 of Crossveinless 2 with BMP-2 allows to combine the weak affinities of two small epitopes and broader binding specificity [44]. Here the local intrinsic disorder in the binding epitope of BMPR-IA allows adaptation of its interface to very different binding partners. Even though the Fab is not a physiological BMP interaction partner, the very same mechanism, by selecting the matching conformation from a pre-existing population, might also explain the promiscuous binding of BMPR-IA to other BMPs, like BMP-6, -7 or GDF-5 [45] or to structurally unrelated molecules like Rgma/Dragon [46]. As flexible loop elements in BMP type I receptors have indeed been shown to modulate promiscuity and specificity [13], [47], further structural studies of protein-protein complexes involving BMPR-IA are necessary to reveal whether and how other binding partners utilize the conformational flexibility of BMPR-IA for recognition and binding to its full extent.

## Supporting Information

Figure S1 (A) B-factor distribution of the heavy chain Cα-atoms of the four AbD1556 molecules in the asymmetric unit of the AbD1556:BMPR-IA crystal. Elevated B-factors (≥ 120Å^2^) are observed for Cα-atoms in the loops of the C_H_-domain, which are not stabilized by crystal-lattice contacts (see Fig S2). (B) As in (A) but for the light chain of the four AbD1556 molecules in the asymmetric unit. (C) As in (A) and (B) but for the Cα-atoms of the BMPR-IA ectodomain of the four complexes AbD1556:BMPR-IA.
(0.63 MB TIF)

Figure S2 Analysis of the crystal lattice contacts of the C_H_ domain of AbD1556 (AbD1556_H_ 1) (A) and BMPR-IA (BMPR-IA4) (B) showing that the elevated B-factors (Fig. S1) observed in these regions correlate with lack of contacts between residues of these areas with symmetry-related molecules. The four complexes AbD1556:BMPR-IA are shown as ribbon plot and color-coded by the B-factor of the backbone (blue indicates B-factors of ≤ 60Å^2^, red indicates B-factors ≥ 120Å^2^), symmetry-related molecules forming the crystal lattice are shown as Cα-trace colored in grey.
(1.73 MB TIF)

Figure S3 Biological activity of the two BMPR-IA binding Fab antibodies AbD1556 and AbD1564. (A) BMP-2 induces the expression of alkaline phosphatase (ALP) in C2C12 cells in a dose-dependent manner. The concentration for half-maximal response (EC_50_) is about 19 ± 1nM. (B) Both Fab AbD1556 and AbD1564 bind to a BMPR-IA epitope that overlaps with BMPR-IA binding to BMP-2 and thus can neutralize BMP-2 activity in the above ALP assay. BMP-2 was added at 20nM and increasing concentrations of AbD1556 or AbD1564 were added. The concentration for half-maximal inhibition is about 90nM for AbD1556 and 60nM for AbD1564.
(0.19 MB TIF)

Figure S4 (A) Ligplot analysis of the interaction of the Fab's AbD1556 V_H_ domain and BMPR-IA. Hydrogen bonds are indicated as green stippled lines, with distances between the acceptor and donor atom shown. The buried surface area upon complex formation is given in Å^2^ next to the residue name. Residues of the Fab are shown with orange lines and annotated with V_H_, residues of BMPR-IA are shown with blue lines and labelled with R. (B) As in (A) but for the interaction of the Fab V_L_ domain and BMPR-IA.
(1.09 MB TIF)

Figure S5 (A) Surface representation of the BMPR-IA binding epitope of AbD1556. The surface is color-coded by amino acid polarity with hydrophobic amino acids (A, C, F, G, H, I, L, M, P, V, W, Y) in dark grey, with acidic residues in red (D, E), basic amino acids marked in blue (K, R) and polar, uncharged residues shown in green. Residues not participating in the binding epitope are shown in lighter colors. (B) As in (A) but for the BMPR-IA binding epitope of BMP-2 (PDB entry 1REW). BMP-2 oriented such that BMPR-IA in the complexes AbD1556:BMPR-IA and BMP-2:BMPR-IA (1REW) are structurally aligned. (C) As in (A) but rotated by about 70° around the x-axis. (D) As in (B) but rotated around the y-axis for about 70°. The top view of the BMPR-IA binding epitopes of AbD1556 (A) and BMP-2 (B) show a seemingly similar distribution of the amino acid chemistry at the binding surface, e.g. a large central hydrophobic patch, surrounding polar or charged residues which (in part) occupy similar positions (e.g. AbD1556 Asp50:BMP-2 Asp53; AbD1556 Trp101:BMP-2 Leu66/Ile62; AbD1556 Arg104:BMP-2 Lys101, etc.). The side view (C,D), however, shows that surface complementarity is rather limited with the curvature of AbD1556 being flat and the BMP-2 surface being highly concave.
(1.87 MB TIF)
