# Supplementary figures and images for: A Selection Fit Mechanism in BMP Receptor IA as a Possible Source for BMP Ligand-Receptor Promiscuity
Source: PLoS One. 2010 Sep 28;5(9):e13049. doi: 10.1371/journal.pone.0013049 (PMC2946932; doi:10.1371/journal.pone.0013049)

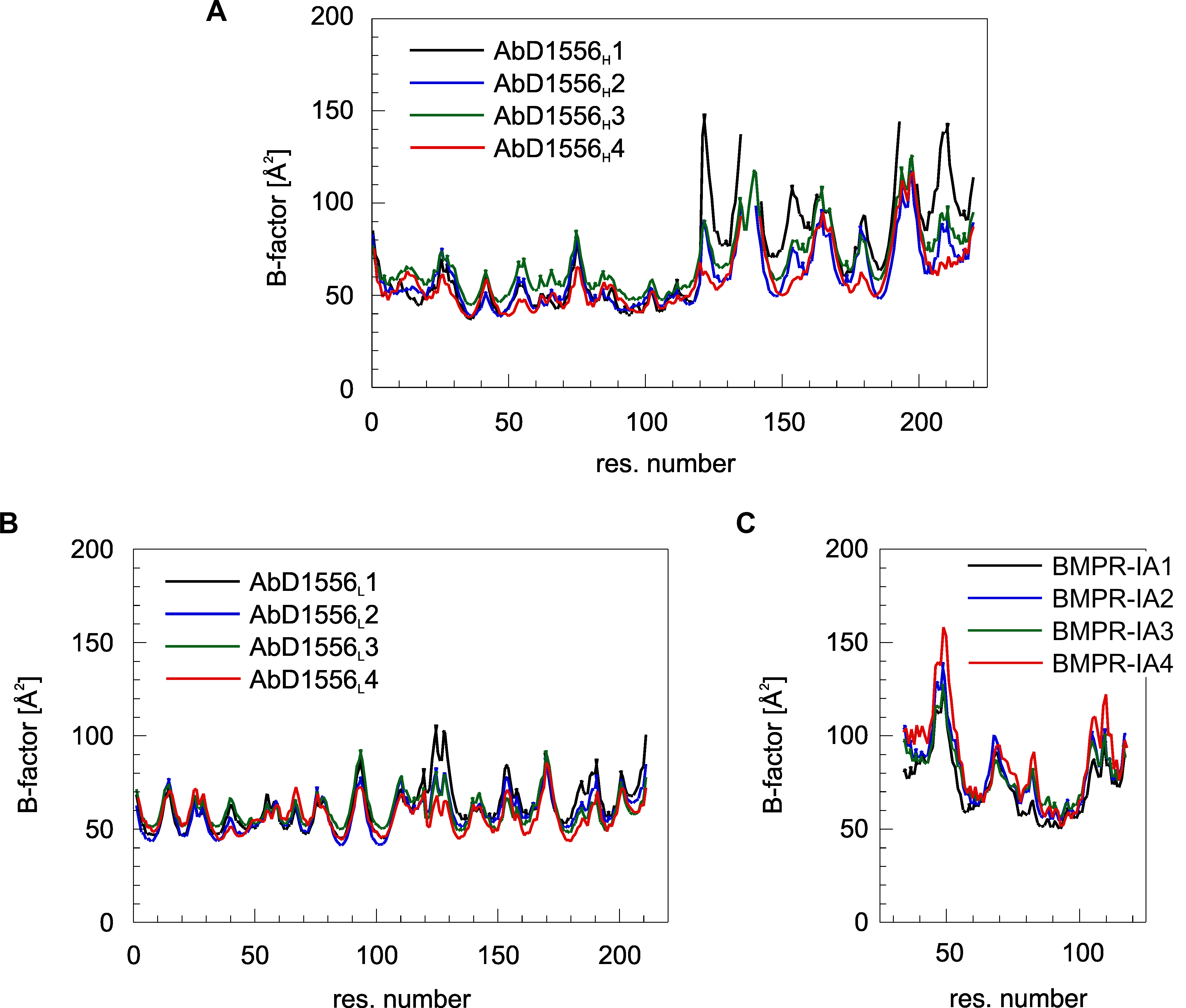

Supplement: Figure S1 — (A) B-factor distribution of the heavy chain Cα-atoms of the four AbD1556 molecules in the asymmetric unit of the AbD1556:BMPR-IA crystal. Elevated B-factors (≥ 120Å2) are observed for Cα-atoms in the loops of the CH-domain, which are not stabilized by crystal-lattice contacts (see Fig S2). (B) As in (A) but for the light chain of the four AbD1556 molecules in the asymmetric unit. (C) As in (A) and (B) but for the Cα-atoms of the BMPR-IA ectodomain of the four complexes AbD1556:BMPR-IA. (0.63 MB TIF) [file pone.0013049.s001.tif]

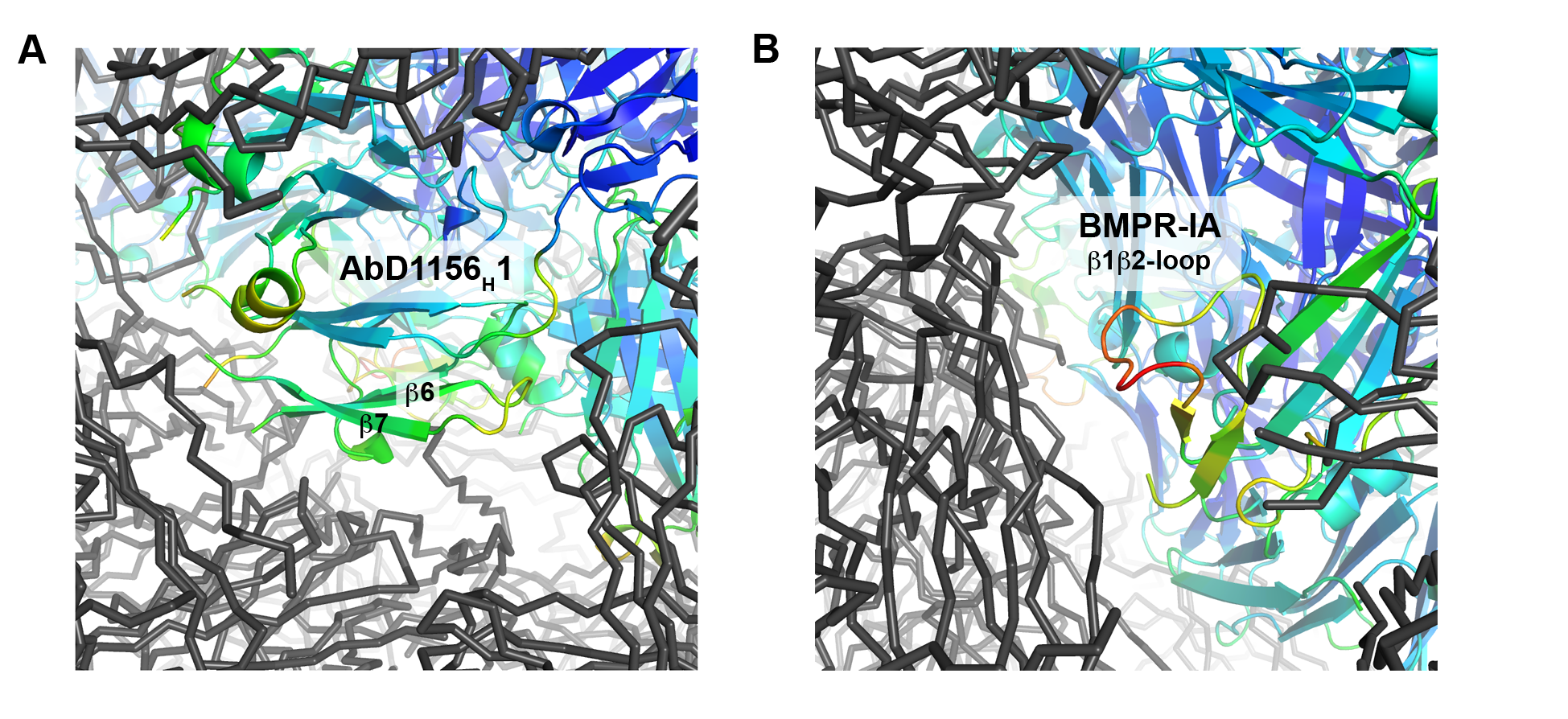

Supplement: Figure S2 — Analysis of the crystal lattice contacts of the CH domain of AbD1556 (AbD1556H 1) (A) and BMPR-IA (BMPR-IA4) (B) showing that the elevated B-factors (Fig. S1) observed in these regions correlate with lack of contacts between residues of these areas with symmetry-related molecules. The four complexes AbD1556:BMPR-IA are shown as ribbon plot and color-coded by the B-factor of the backbone (blue indicates B-factors of ≤ 60Å2, red indicates B-factors ≥ 120Å2), symmetry-related molecules forming the crystal lattice are shown as Cα-trace colored in grey. (1.73 MB TIF) [file pone.0013049.s002.tif]

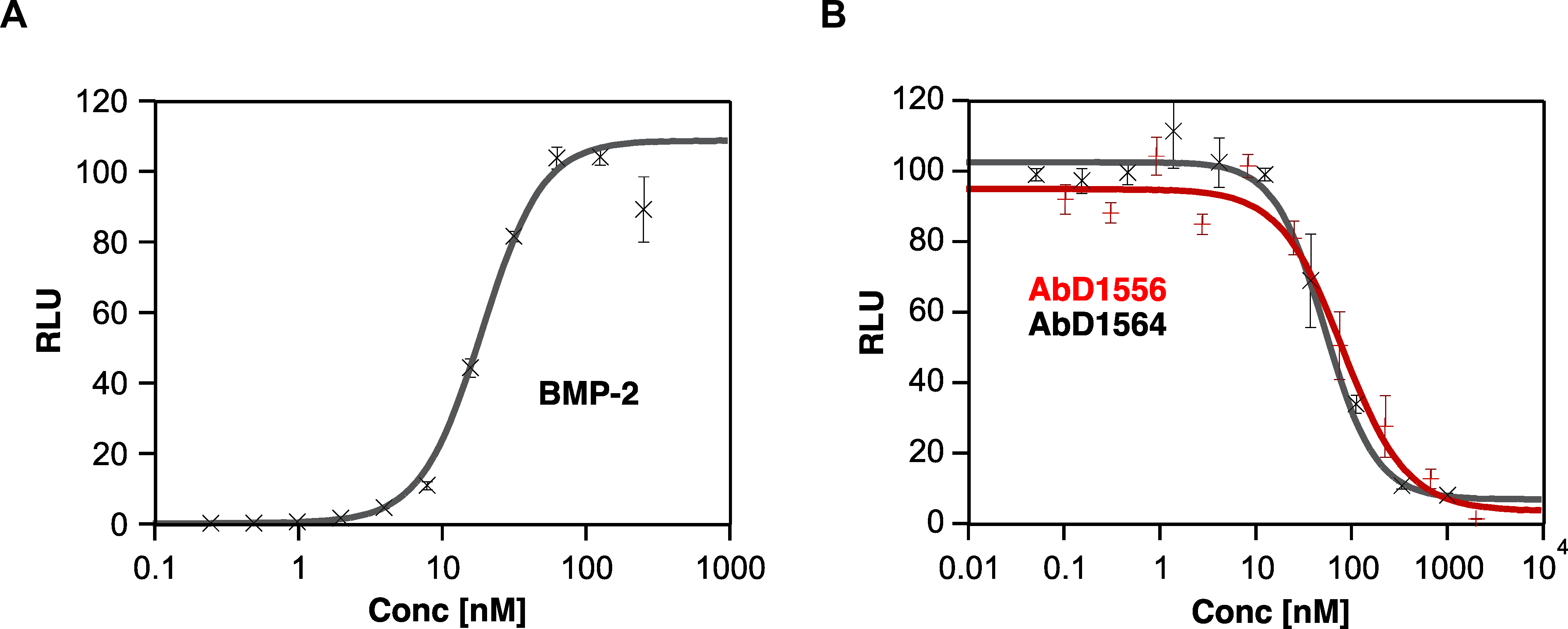

Supplement: Figure S3 — Biological activity of the two BMPR-IA binding Fab antibodies AbD1556 and AbD1564. (A) BMP-2 induces the expression of alkaline phosphatase (ALP) in C2C12 cells in a dose-dependent manner. The concentration for half-maximal response (EC50) is about 19 ± 1nM. (B) Both Fab AbD1556 and AbD1564 bind to a BMPR-IA epitope that overlaps with BMPR-IA binding to BMP-2 and thus can neutralize BMP-2 activity in the above ALP assay. BMP-2 was added at 20nM and increasing concentrations of AbD1556 or AbD1564 were added. The concentration for half-maximal inhibition is about 90nM for AbD1556 and 60nM for AbD1564. (0.19 MB TIF) [file pone.0013049.s003.tif]

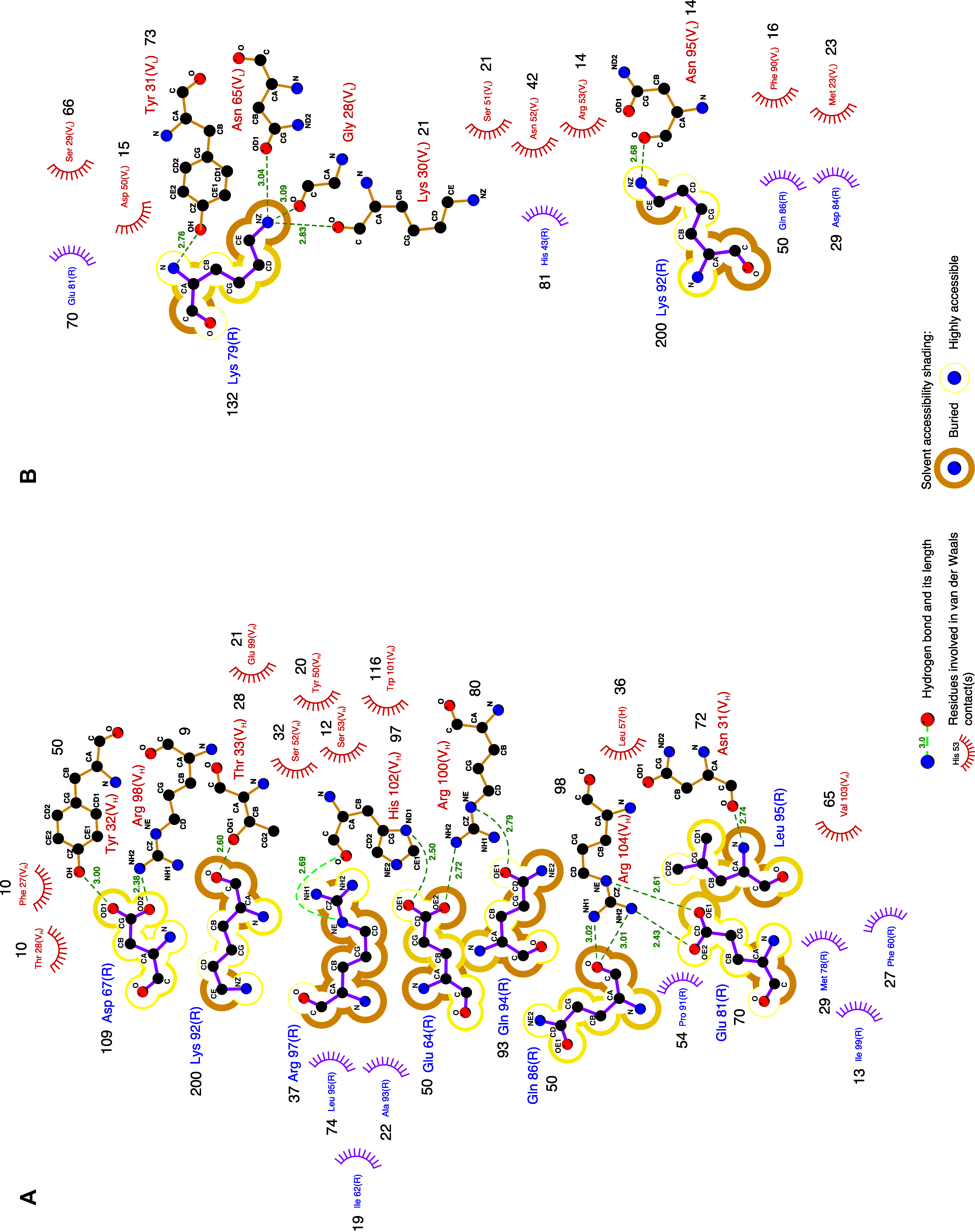

Supplement: Figure S4 — (A) Ligplot analysis of the interaction of the Fab's AbD1556 VH domain and BMPR-IA. Hydrogen bonds are indicated as green stippled lines, with distances between the acceptor and donor atom shown. The buried surface area upon complex formation is given in Å2 next to the residue name. Residues of the Fab are shown with orange lines and annotated with VH, residues of BMPR-IA are shown with blue lines and labelled with R. (B) As in (A) but for the interaction of the Fab VL domain and BMPR-IA. (1.09 MB TIF) [file pone.0013049.s004.tif]

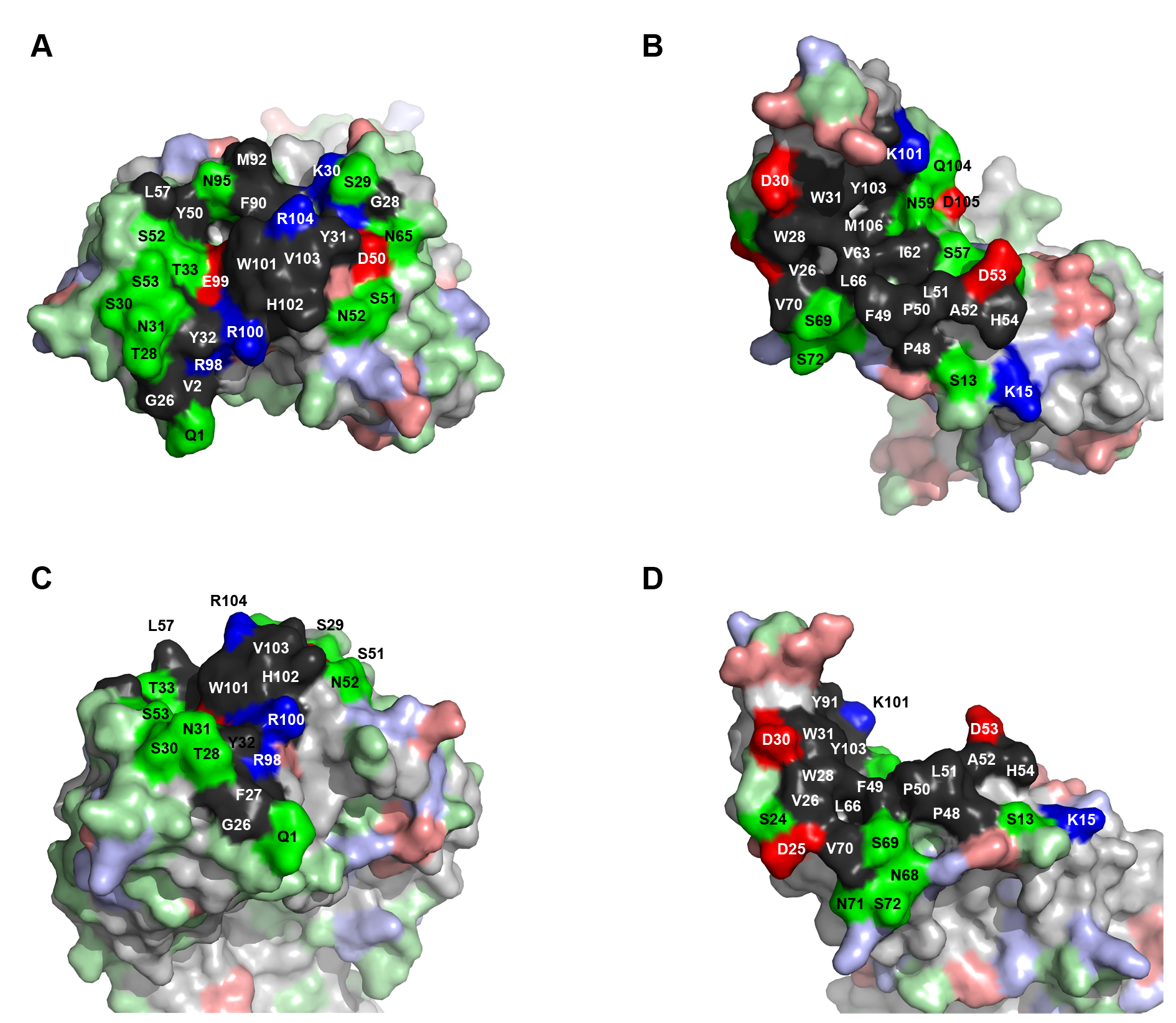

Supplement: Figure S5 — (A) Surface representation of the BMPR-IA binding epitope of AbD1556. The surface is color-coded by amino acid polarity with hydrophobic amino acids (A, C, F, G, H, I, L, M, P, V, W, Y) in dark grey, with acidic residues in red (D, E), basic amino acids marked in blue (K, R) and polar, uncharged residues shown in green. Residues not participating in the binding epitope are shown in lighter colors. (B) As in (A) but for the BMPR-IA binding epitope of BMP-2 (PDB entry 1REW). BMP-2 oriented such that BMPR-IA in the complexes AbD1556:BMPR-IA and BMP-2:BMPR-IA (1REW) are structurally aligned. (C) As in (A) but rotated by about 70° around the x-axis. (D) As in (B) but rotated around the y-axis for about 70°. The top view of the BMPR-IA binding epitopes of AbD1556 (A) and BMP-2 (B) show a seemingly similar distribution of the amino acid chemistry at the binding surface, e.g. a large central hydrophobic patch, surrounding polar or charged residues which (in part) occupy similar positions (e.g. AbD1556 Asp50:BMP-2 Asp53; AbD1556 Trp101:BMP-2 Leu66/Ile62; AbD1556 Arg104:BMP-2 Lys101, etc.). The side view (C,D), however, shows that surface complementarity is rather limited with the curvature of AbD1556 being flat and the BMP-2 surface being highly concave. (1.87 MB TIF) [file pone.0013049.s005.tif]
